# Surface Curvature Matters in Electrochemical Reactions

**DOI:** 10.1021/acscentsci.3c01637

**Published:** 2024-01-10

**Authors:** Cuiling Li, Yusuke Yamauchi

**Affiliations:** † CAS Key Laboratory of Bio-Inspired Materials and Interface Science, Technical Institute of Physics and Chemistry, Chinese Academy of Sciences, Beijing 100190, China; ‡ Australian Institute for Bioengineering and Nanotechnology (AIBN), The University of Queensland, Brisbane 4072, Australia; § Department of Materials Process Engineering, Graduate School of Engineering, Nagoya University, Furo-cho, Chikusa-ku, Nagoya, Aichi 464-8603, Japan

**Figure 1:**
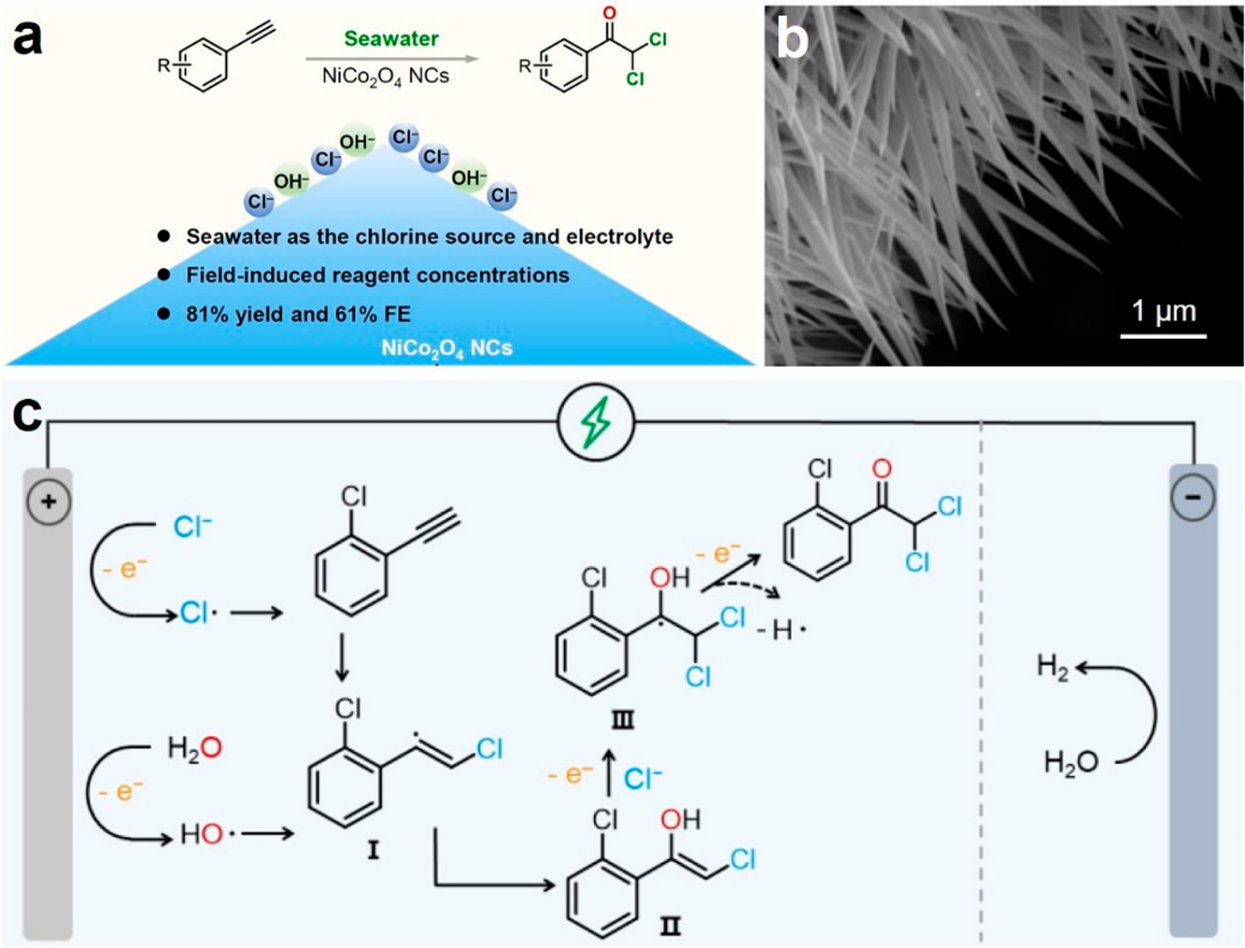
(a) Advantages and (c) proposed possible reaction mechanism for the electrooxidation of ethynylbenzenes to α,α-dichloketone catalyzed by NiCo_2_O_4_ nanocones. (b) SEM image of the NiCo_2_O_4_ nanocones. Reproduced with permission from ref (1). Copyright 2023 American Chemical Society.

Electrochemical synthesis, which is driven by electrons generated at the electrode surface to initiate reactions, has emerged as a promising approach in organic synthesis due to its environmentally friendly and efficient features. As electron transfer occurs on the electrode surface, the choice of electrode, including its composition, morphology, and geometry, significantly influences the reactions. In this issue of *ACS Central Science*, Zhang, Wu, and co-workers report the electrooxidization of ethynylbenzenes to α,α-dichloketone by directly utilizing seawater as the chlorine source and electrolyte solution, achieving a high performance of 81% yield, a 61% Faradaic efficiency, and a yield rate of 44.2 mmol g^–1^_cat._ h^–1^. The high-curvature NiCo_2_O_4_ nanocones are critical for realizing this performance because they enhance the local electric field to enrich reactant ions near the electrode surface. This acceleration facilitates the activation of Cl^–^ and H_2_O in the electrolyte to form Cl̇ and OḢ radicals. These radicals then react with ethynylbenzenes through a Cl̇-radical-triggered Cl̇ and OḢ radical addition pathway (Figure 1).^1^

Considering the positive effect of an electric field on enhancing the activity and selectivity of chemical reactions, nanostructures that can intrinsically evoke an electric field have sparked significant research interest. Among all of the investigated nanoarchitectures, those with high-curvature surfaces have demonstrated extreme importance in inducing an electric field for enhanced catalytic performance. This is achieved by altering the reaction thermodynamics and enriching ions/reagents at the catalyst surface. A notable example is high-curvature Au tips, which create a large local electric field, concentrating K^+^ ions and CO_2_ molecules at the catalyst surface, thereby enhancing the reaction rates of electrochemical CO_2_ reduction.^2^ In addition to the above-mentioned nanostructures with sharp-tips (e.g., nanoneedles and nanocones), porous materials exhibit tunable curvature of concave surfaces by rationally controlling pore sizes.^3^ Pore sizes have been widely controlled in the range from several nanometers to hundreds of nanometers using different porogens (micelles, mesoporous silica, and polystyrene/silica spheres) through either a soft templating method or a hard templating method, suggesting a broad range for regulating surface curvature.^4−7^ Furthermore, a more convincing feature of the concave curvature in porous materials is that plenty of catalytically active sites, including unsaturated atomic structures and/or lattice strains, are formed depending on the surface curvature.^7,8^ In particular, porous copper-based materials have achieved outstanding electrochemical CO_2_-to-multicarbon product (CO_2_-to-C_2+_) conversion by capitalizing on the merits of the steric confinement effect of the interconnected porous channels.^9^

The control of surface curvature stimulates intrinsic physical and chemical characteristics essential for enhanced electrochemical performance, offering promise for targeted synthesis under moderate conditions. The team lead by Zhang demonstrated commendable results in the electrooxidation of ethynylbenzenes to α,α-dichloketone using high-curvature NiCo_2_O_4_ nanocones as electrocatalysts. This synthetic strategy avoids the use of corrosive chlorine, harsh reaction conditions, and excessive electrolytes, as used in conventional synthetic procedures. Further investigations into creating nanostructured catalysts with curvature control may lead to superior electrochemical performances and more sophisticated electrochemical synthesis techniques.
